# Mechanisms of Epithelial Immunity Evasion by Respiratory Bacterial Pathogens

**DOI:** 10.3389/fimmu.2020.00091

**Published:** 2020-02-11

**Authors:** Lokesh Sharma, Jingjing Feng, Clemente J. Britto, Charles S. Dela Cruz

**Affiliations:** ^1^Section of Pulmonary, Critical Care and Sleep Medicine, Yale University School of Medicine, New Haven, CT, United States; ^2^Department of Respiratory Medicine, The Fifth People's Hospital of Shanghai, Fudan University, Shanghai, China

**Keywords:** bacterial dissemination, host pathogen interaction, epithelial immunity, pathogen evolution, lung epithelium

## Abstract

Bacterial lung infections are major healthcare challenges killing millions of people worldwide and resulting in a huge economic burden. Both basic and clinical research have elucidated host mechanisms that contribute to the bacterial clearance where an indispensable role of immune cells has been established. However, the role of respiratory epithelial cells in bacterial clearance has garnered limited attention due to their weak inflammatory or phagocytic ability compared to immune cells such as macrophages and neutrophils. These studies often underappreciate the fact that epithelial cells are the most abundant cells in the lung, not only serving as building blocks but also providing immune protection throughout the lung. Epithelial cells function either independently to eradicate the pathogen or communicate with immune cells to orchestrate pathogen clearance. The epithelial cells have multiple mechanisms that include mucus production, antimicrobial peptide production, muco-ciliary clearance, and phagocytosis, all of which contribute to their direct antibacterial function. Secretion of cytokines to recruit immune cells and potentiate their antimicrobial activities is a pathway by which the epithelium contributes to bacterial clearance. Successful pathogens outsmart epithelial resistance and find a way to replicate in sufficient numbers to establish infections in the airway or lung epithelial surfaces. In this mini-review, we discuss evidences that establish important roles for epithelial host defense against invading respiratory bacterial pathogens and demonstrate how pathogens outsmart these epithelial immune mechanisms to successfully establish infection. Finally, we discuss briefly how to boost epithelial immunity to improve outcomes in bacterial lung infections.

## Epithelial Immunity in the Lung

The lung surface area of more than 50 m^2^ consists of epithelial monolayers which cover the upper and lower airways as well as lung alveoli (1). The large epithelial surfaces are continuously exposed to particles, pollutants, and microbes. It is not surprising that the lung epithelium has a well-developed immunity to fend off these microbes. Different subsets of lung epithelium are equipped with their unique antimicrobial mechanisms (2–4). While nasal epithelium is known to secrete large amounts of antimicrobial peptides (AMPs) and type II pneumocytes secrete surfactant proteins, both of these are host protective mechanisms against bacterial infections. Ciliated airway epithelium mechanically removes invading pathogens with the help of mucus secreted by the secretory epithelium. If the epithelium fails to limit the invading pathogens itself, the epithelium signals to seek the help of resident immune cells such as alveolar macrophages or to recruit immune cells to help eradicate the invading pathogen, which often comes at a cost of tissue injury, including to the epithelium (5, 6). Lung epithelium also contributes to the removal of dead inflammatory cells to ensure restoration of lost lung function post infection. However, pathogens have developed multiple mechanisms to avoid various epithelial resistance mechanisms. Respiratory pathogens discussed here include clinically relevant pathogens such as *Pseudomonas aeruginosa* (infects patients with chronic lung diseases such as cystic fibrosis and COPD) (7), *Streptococcus pneumoniae* (affects healthy people and causes post-viral bacterial infections) (8), *Staphylococcus aureus* (major cause of hospital-acquired pneumonia) (9), and *Klebsiella pneumoniae* (opportunistic pathogen with high antimicrobial resistant) (10).

Antimicrobial functions of the lung epithelium can be divided into two main categories: direct killing and indirect control of lung infections.

### Direct Killing

**Figure 1:**
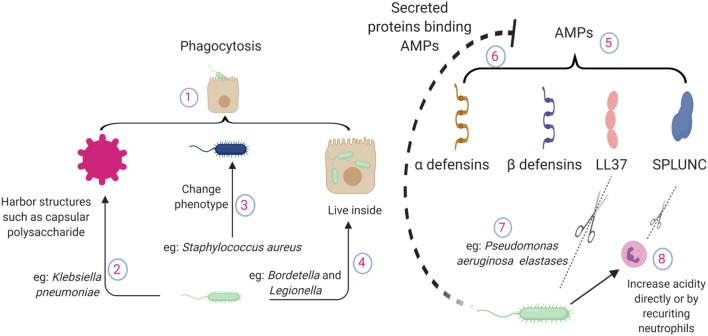
Pathogen mechanisms to circumvent direct killing of bacteria by lung epithelial cells. (1) Epithelial cells possess phagocytic ability. (2) Bacteria harbor structures to evade epithelial phagocytosis, such as capsular polysaccharides of *Klebsiella pneumoniae*. (3) Altered phenotypes in pathogens cause chronic infections e.g., small colony variant of *Staphylococcus aureus*. (4) Pathogens can live inside the lung epithelium e.g., *Bordetella or Legionella*. (5) Epithelium releases antimicrobial peptides during lung infections, which bind to the pathogen surface and neutralize the pathogen. (6) During inflammation, a large number of proteins are accumulated in the lung. AMPs interact with other proteins and lose their antimicrobial potency. (7) *P. aeruginosa* elastase breaks down human LL37 in a dose dependent manner. (8) Pathogen recruited neutrophils contribute to the acidity. SPLUNC loses its function in an acidic environment; also, neutrophil elastases can break down SPLUNC. AMPs, antimicrobial proteins and peptides; SPLUNC, short palate lung and nasal epithelium clone 1.

Killing of the invading pathogen is the most effective strategy to remove the invading pathogen, however the primary aim of the epithelium is to survive the infection (Figure 1). To avoid unnecessary inflammatory damage, which may be unavoidable consequence of the pathogen killing, the host epithelium tolerates commensal microbes or colonizing pathogens, which are present at higher density especially in the upper respiratory tract (11). However, an appropriate level of active epithelial immunity is required to maintain the colonization status of these pathogens. Any impairment in the epithelial immunity can lead to infection from colonizing pathogens, as seen during influenza infection, where nasally colonized *Streptococcus* invades and establishes a lower respiratory tract infection (12). Multiple mechanisms have been attributed to influenza enhanced susceptibility including a damaged epithelial barrier, increased micronutrition due to cell death and decreased surfactant proteins (13). Other colonizing pathogens such as *Staphylococcus aureus* show similar infecting mechanisms post influenza, where colonizing pathogens are often associated with the disease. The detailed mechanisms of colonization are discussed in other reviews (14). The epithelium has multiple tools to limit the growth of pathogens or to remove them completely from the host.

#### Phagocytosis

Phagocytosis is the main mechanism of bacterial killing used by professional phagocytes such as macrophages and neutrophils. However, epithelial cells also possess significant phagocytic ability, which is often referred to as “internalization” against a wide range of bacterial pathogens (15, 16). There are major differences in the capacity and mechanisms of phagocytosis among the professional phagocytes and epithelial cells. Professional phagocytes like macrophages actively phagocytose the pathogens by recognizing them using its pattern recognition receptors, where antibody mediated opsonization plays a major role in the process (17). On the other hand, epithelial phagocytosis is relatively low and often initiated by the pathogen. Pathogens use two distinct mechanisms to enter the epithelial cells: “zipper” and “trigger” mechanisms (18). In the “zipper” mechanism, pathogen attachment induces rearrangement of actin cytoskeleton and the resultant internalization. In the “trigger” method, the pathogen secretes soluble factors that cause actin fiber rearrangement to form a phagocytic cup and subsequent internalization of the pathogen. However, the epithelium expresses various pattern recognition receptors that actively recognize the invading pathogen and allow them to respond to the pathogen by releasing cytokines and chemokines. A wide range of TLRs and nod like receptors have been detected on lung epithelium on both the surfaces as well as within the cell (19–21). Although epithelial cells have lower phagocytic activity per cell compared to the professional phagocytes, given the number of epithelial cells in the lung compartment and the surface area covered by these cells, their phagocytic activity provides significant contribution to pathogen clearance.

Phagocytosis allows digestion of the bacterial components within the cell and thus limits inflammatory signaling arising from extracellular bacterial killing. Antibiotic induced bacterial killing has been shown to be highly inflammatory (22). Phagocytic ability of the epithelium also helps maintain the homeostatic lung environment, which is disrupted in the aftermath of an infectious injury by an abundance of dead cells and cellular debris from various cells, including the epithelium. Epithelial phagocytosis of apoptotic cells is dependent on small GTPase Rac-1, which allows the epithelium to secrete anti-inflammatory cytokines upon phagocytosis of cell debris to inhibit inflammatory recruitment and restore homeostasis (23).

##### How pathogens circumvent phagocytic ability

Epithelial-mediated phagocytosis is dependent on various host and pathogen factors. Pathogens often harbor structures on their surface to evade epithelial phagocytosis as seen in *Klebsiella*, where the amount of capsular polysaccharide is inversely related to epithelial internalization. As expected, bacterial strains lacking capsular polysaccharides are effectively cleared by the lung epithelium (24). *Pseudomonas* forms multicellular complexes known as biofilms, which are enormous in size, to prevent phagocytosis (25, 26). Avoiding epithelial phagocytosis may even provide *Pseudomonas* an advantage by dysregulating the inflammatory response and causing systemic sepsis that is detrimental to the host but supports bacterial growth (27). Some pathogens have developed ways to reside within the epithelium, such as *Bordetella* which uses host tubulin activity to enter the epithelial cells. This entry provides protection not only against antibiotic treatment but also against host antimicrobial peptides (28). Pathogens also avoid fusion with acidic lysosomes, which are filled with enzymes that can lyse the bacterial cell wall by manipulating cellular trafficking. Similarly, highly pathogenic strains of *Legionella* reside and replicate in the lung epithelium in addition to replicating within macrophages, which are normal targets of *Legionella* (29). Pathogens also alter their phenotype to adjust themselves within the epithelium to cause chronic infections. A small colony variant of *Staphylococcus aureus* often persists intracellularly including in the epithelium due to its ability to exert minimal inflammatory response compared to wild type strains (30). These examples demonstrate a wide array of pathogen mechanisms to outsmart epithelial phagocytosis, which contributes to the disease pathogenesis.

#### Antimicrobial Proteins and Peptides (AMPs)

The lung epithelium is a primary source of many AMPs released during lung infection. AMPs are charged peptides that can bind to the pathogen surface and kill them. These AMPs are separate from complement pathways, which primarily “tag” the pathogen to be identified by professional phagocytes, although some evidences of direct killing of pathogens by complements has been reported (31).

Defensins are a group of AMPs, of which β-defensins are almost exclusively secreted by the lung epithelium (32). Inflammatory cytokines produced during infection can lead to the release of AMPs (33, 34). The role of AMPs has been well established whereby they kill a wide range of pathogens including *E. coli, Pseudomonas* and *Haemophilus influenzae* in a salt dependent manner (34, 35). These antibacterial effects are confirmed in a mouse model of *H. influenzae*, where β-defensin deficiency impaired bacterial clearance (36). Over-expression of β-defensin 2 in a porcine model has been shown to increase the clearance of *Actinobacillus pleuropneumoniae* (37).

Cathelicidins, another important epithelial AMPs, are cleaved into their mature form LL37. Unlike β-defensins, only a single cathelicidin peptide has been found in the lungs of various species, including humans. The positively charged LL37 is secreted by lung epithelial cells (38) and has bactericidal activity against a variety of lung pathogens such as *P. aeruginosa* and *S. aureus* (39, 40). Besides direct antibacterial activity, LL37 also promotes bacterial phagocytosis in macrophages (41), indicating AMPs serve as important communicators between the epithelial and immune cells.

More recently, short palate lung and nasal epithelium clone 1 (SPLUNC) has been identified as an important AMP expressed in the upper airways and has a binding affinity for lipopolysaccharides (LPS) (42). SPLUNC has been shown to protect mice from infection with respiratory pathogens such as *P. aeruginosa* and *Mycoplasma* (43, 44). Furthermore, this study demonstrated that there might be a positive feedback mechanism between various AMPs since deficiency of SPLUNC1 led to decreased expression in LL37.

##### How pathogens evade AMP mediated death

Successful pathogens often develop strategies to overcome AMP-mediated host defense. These strategies include cleavage of AMPs by secreted proteases such as aureolysin and V8 protease from *S. aureus* (45). *P. aeruginosa* elastase can effectively break down LL37 (46) and SPLUNC (47). Given the wide substrate range, it is very likely that various AMPs are susceptible to host neutrophil elastase mediated cleavage and subsequent loss of antibacterial activity, as neutrophil elastase levels are significantly increased during bacterial infections (48).

During inflammation, a large number of host and pathogen proteins/products are accumulated in the lung airspace. Given the charged nature of AMPs, they easily interact with other charged molecules and lose their antimicrobial potency (49). Pathogens also modulate the lung environment to decrease the toxicity of AMPs. For example, the antimicrobial properties of SPLUNC are lost in the airways in cystic fibrosis due to its acidic environment. Pathogens can directly create an acidic environment by changing their metabolic activity or by recruiting metabolically active immune cells such as neutrophils (50).

Pathogens also shed components that actively bind to AMPs and prevent their binding to the bacterial surface to avoid AMP mediated toxicity. LPS, a part of the bacterial outer membrane in gram negative pathogens, are shed during infection. β-defensin binds to LPS and blocks its signaling. At the same time, LPS effectively block the antimicrobial activity of AMPs by direct binding (51). The capsule polysaccharide from *K. pneumoniae* has been shown to act as a bacterial decoy for AMPs secreted by the host to abrogate antimicrobial capacity (52).

### Indirect Control of Lung Infections

**Figure 2:**
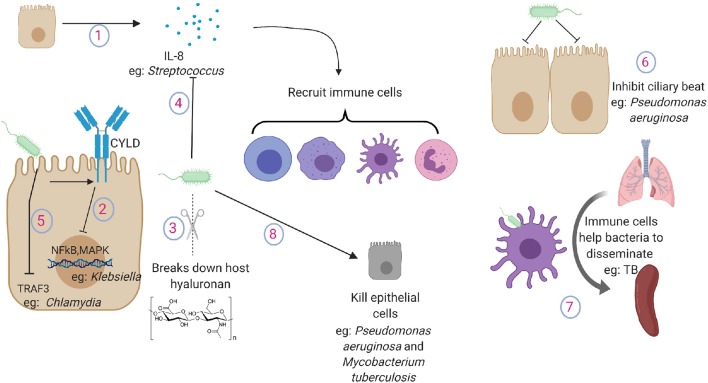
Pathogen tactics to avoid indirect killing during lung infections. (1) Lung epithelium secrete cytokines and chemokines to recruit immune cells in the lung. (2) Klebsiella inhibits the activation of NFkB and MAPK pathway by promoting the expression of ubiquitinating enzyme CYLD in lung epithelial cells. (3) Gram positive pathogens break down host hyaluronan. (4) *Streptococcus* cleaves and inactivates IL-8. (5) Chlamydia targets TRAF3 in the epithelial cells. (6) *Pseudomonas aeruginosa* inhibits ciliary beat function. (7) Macrophages and dendritic cells carry TB from the lung to secondary lymphoid organs. (8) Pathogens induce damage to lung cells or to the extracellular matrix. IL-8, interleukin-8; NFkB, nuclear factor kappa B; MAPK, mitogen activated protein kinase; TRAF3, TNF receptor-associated factor 3.

Despite having well-developed mechanisms to prevent bacterial invasion, the epithelium often fails to completely eradicate the pathogens. In these situations, the epithelium often collaborates with other cells to ensure proper removal of invading pathogens. In cases where removing the pathogen completely seems impossible, the epithelium ensures that the infection remains localized and does not spread systemically. To perform these indirect functions, the epithelium communicates with other cells either by direct contact or by releasing soluble factors like cytokines and chemokines. Mechanisms of indirect pathogens control include the following: (see Figure 2).

#### Recruitment of Immune Cells

If the lung epithelium fails to control the growth of invading pathogens, it recruits immune cells to participate in the bacterial removal process. To sense pathogen, pattern recognition receptors are expressed on both apical and basolateral surfaces of the epithelium (20). Upon recognition of the pathogens, these cells secrete cytokines and chemokines to increase the expression of complementary adhesion molecules on the vascular endothelium and immune cells. The epithelium itself also expresses adhesion molecules such as ICAM-1 and VCAM1 (53) to facilitate the recruitment of immune cells in the lung. Epithelial-macrophage interactions can further amplify the secretion of cytokines to mount a robust inflammatory response; for example, epithelial release of CXCL8 can be enhanced by incubation with macrophages or macrophage supernatants (54). Upon recruitment, immune cells eliminate the pathogens by phagocytosis or extracellular killing by neutrophils and macrophage extracellular traps (55–57). Immune cells are highly effective in pathogen clearance but may cause lung injury by proteolytic enzymes contained within the immune cells. Upon pathogen clearance, the epithelium also contributes to clearance of the infiltrated immune cells. Conventionally, macrophages have been credited to clear the dead cells present in the lung post inflammatory response; however, recent studies have also implicated important roles of the lung epithelium in clearing the dead cells and cellular debris from the lung and preventing persistent inflammation (58). Blocking epithelial phagocytosis by inducible mutation of small GTPase Rac1 leads to persistent lung inflammation (23).

##### How pathogens blunt inflammatory responses

As inflammation is essential for effective clearance of invading bacterial pathogens, the pathogens can evade this inflammation by either suppressing the inflammatory response or by causing excessive inflammatory response. *K. pneumoniae* lung infections often induce very limited early inflammatory response, and it successfully disseminates across organs before inducing a robust inflammatory response (59). *Klebsiella* inhibits the activation of NFkB and MAPK pathway by promoting the expression of ubiquitinating enzyme CYLD in lung epithelial cells (60). *Streptococcus* secretes hyaluronidases that break down host hyaluronan into small components that inhibit not only inflammatory host hyaluronan fragments but also other TLR ligands such as LPS (61). Epithelial cells can recognize the pathogens and secrete IL-8, which is a potent chemokine in the lungs (62). *Streptococcus* species that cause aggressive disseminating disease have developed mechanisms by which they can cleave and inactivate IL-8 to blunt the inflammatory response through cell envelope proteinase (63). *Chlamydia pneumoniae* manipulates epithelial immunity by targeting TRAF3 in the epithelial cells and thus impairing TRAF3 mediated immunity (64). In contrast, some pathogens can use the presence of inflammatory cytokines as growth factors and survive excessive inflammation (65).

#### Prevent Bacterial Dissemination

The lung epithelium is a continuous barrier between the environment and circulation. This barrier ensures that pathogens in the lung do not achieve access to the circulation. Pathogens often strive to reach the blood circulation, likely due to the high amount of nutrients present in the blood. It is well documented that systemic spread of lung infection results in higher mortality compared to localized lung infection (66, 67).

The tight junctions between the lung epithelium are maintained by various junction proteins such as occludin family members and ZO-1 (68). Various enhancements in the lung epithelium increases the effectiveness of the barrier. These include muco-ciliary clearance in the upper airways (69) and surfactant proteins secreted by type II pneumocytes, which can promote bacterial killing including *Klebsiella* (70). Together these physical and biochemical structures ensure that lung infections do not extend outside the lung compartment.

##### How pathogens circumvent the epithelial immunity to disseminate

Ciliary beat function serves as one of the first physical defenses to prevent the pathogen from reaching the lung and subsequently causing systemic infection. It has been shown that virulence factors from *P. aeruginosa* can significantly inhibit ciliary beat, possibly allowing the bacteria to stick to the lung epithelium (71). Despite maintaining the uniform monolayer with sealed tight junctions, the epithelium provides regulated passage to immune cells such as those recruited during infections. This passage is provided by expression of complementary adhesion molecules on both immune and epithelial cells (72). Pathogens often take advantage of this host mechanism to spread by hiding within the immune cells. Antigen presenting cells such as macrophages and dendritic cells are well known to sense the pathogen and leave the lung compartment to present the antigen to lymphoid organs. However, recent evidence suggests that neutrophils also leave the lung and return to the bone marrow through the circulation once the inflammation is resolved (73). Macrophages and dendritic cells are believed to carry *Mycobacteria tuberculosis* from the lung to secondary lymphoid organs such as spleen (74). Other mechanisms of bacterial dissemination across the lung epithelium such as “ejectosomes” observed in amoeba have not yet been described in mammals (75). Pathogens also use TLR pathways to downregulate expression of tight junction protein claudins 7 and 10, which are essential to maintain intercellular junctions (76). Inhibition of claudins allows the pathogen to escape the lung by increasing epithelial permeability and causing systemic disease.

Various pathogens also induce damage to the lung structure by either directly killing epithelial cells or by digesting the extracellular matrix. These effects can be directly caused by pathogens or by pathogen induced host responses (77). *P. aeruginosa* has been shown to inject Exos, a potent toxin, using its type III secretion system to induce localized epithelial cell death to facilitate bacterial dissemination (78). Pore forming toxins secreted by a wide range of pathogens have also been shown to cause necroptotic cell death in the lung epithelium (79). Damage to the lung epithelium by other exposures such as hyperoxia or exposure to environmental particles increases the bacterial dissemination for *P. aeruginosa* (80). *M. tuberculosis* can directly cause epithelial death by secreting virulence factors such as ESAT6, leading to its dissemination (81).

## Boosting Epithelial Immunity

Antibiotics remain the current first line therapy for bacterial infections, but their efficacy is being challenged with current rising of antimicrobial resistance (82). Given the urgency, it is essential to develop alternatives to treat lung infections. As the epithelium provides important host defense, it is logical to boost their function to improve outcomes during infections. Boosting the epithelial host defense can provide an important immunomodulatory strategy, especially in patients who have either a limited number of immune cells (genetic or acquired immunodeficiency, chemotherapy), a defect in immunity derived particularly from defects in the lung epithelium (such as CF), a recipient of lung transplant (where immunosuppression is required to prevent lung rejection) or those with extremes of the age. Boosting epithelial immunity can not only prevent the collateral damage that can be caused by recruited immune cells but also circumvent the need to remove the immune cells from tissue once the invading bacteria is cleared.

A large number of mouse studies have demonstrated the usefulness of promoting epithelial resistance to help the host clear the invading pathogens. These strategies involve stimulation with TLR ligands. These include single reagents such as flagellin (for TLR5) (83), poly I:C (84), aminoalkyl glucosaminide phosphate (synthetic TLR4 ligand) (85), CpG ODN (TLR 9 ligand) (86) or a combination of two or more ligands such as Pam2CSK4 + ODNM62 (87). Possible mechanisms of this trained immunity include early recruitment of immune cells (88) and promoting epithelial resistance by epigenetic changes (89) or promoting reactive oxygen species production (90).

Although these strategies have been highly successful in the mouse models, their use in susceptible human populations still needs to be confirmed. Major challenges in their success remain to be their tolerability in subjects and their provision of sufficient protection in the lungs against these infections.

In summary, the epithelium provides an array of host defense mechanisms to prevent and control bacterial lung infections. However, pathogens often circumvent these mechanisms to establish successful infections. Our aim should be to improve the epithelial host defense to boost host immunity and improve survival in lung infections.

## Author Contributions

LS drafted the manuscript. JF prepared the figures. CB Provided critical comments. CD revised the manuscript and provided critical comments.

### Conflict of Interest

The authors declare that the research was conducted in the absence of any commercial or financial relationships that could be construed as a potential conflict of interest.
